# Post-procedural Wound Infections by Mycobacterium fortuitum: A Series of Seven Cases From Eastern India

**DOI:** 10.7759/cureus.117663

**Published:** 2026-10-08

**Authors:** Diptanu Majumder, Soma Jana, Debjani Das, Mrinmoy Chaterjee, Niladri Das

**Affiliations:** 1 Department of Microbiology, Manipal-Tata Medical College, Jamshedpur, IND; 2 Department of Microbiology, All India Institute of Medical Sciences, Kalyani, Kalyani, IND

**Keywords:** bactalert mp, chronic wound infection, mycobacterium fortuitum, nontuberculous mycobacteria (ntm), ntm culture, post operative infection, rapidly growing mycobacteria (rgm), surgical site infection

## Abstract

Background

Rapidly growing nontuberculous mycobacteria (NTM), particularly *Mycobacterium fortuitum*, are increasingly recognized as causes of chronic post-procedural skin and soft tissue infections. However, delayed clinical suspicion and limited microbiological investigations often result in underdiagnosis, especially in resource-limited settings. We describe the clinical, microbiological, and therapeutic characteristics of a series of patients with post-procedural *M. fortuitum* infection from Eastern India.

Methods

This cross-sectional observational study was conducted at a tertiary care teaching hospital in Eastern India between September 2024 and August 2025. Seven patients with culture-confirmed *M. fortuitum* infection were subsequently described as a case series. Clinical specimens were processed for Ziehl-Neelsen staining, routine bacterial culture, and mycobacterial culture using the BacT/ALERT MP liquid culture system and Middlebrook medium. Species identification was done using matrix-assisted laser desorption/ionization time-of-flight mass spectrometry (MALDI-TOF MS). Clinical presentation, microbiological findings, treatment, and outcomes were analysed descriptively.

Results

*Mycobacterium fortuitum *was isolated from seven of the clinically suspected cases. The mean patient age was 39 years, and six (85.7%) were female. Five patients (71.4%) had undergone their primary procedure at peripheral nursing homes. Patients presented with chronic non-healing wounds, persistent serous or seropurulent discharge, delayed wound healing, and poor response to conventional antibacterial therapy. Direct Ziehl-Neelsen staining was positive in two (28.6%) cases, whereas all isolates were recovered by BacT/ALERT MP liquid culture within 4-11 days and identified as *M. fortuitum* by MALDI-TOF MS. All patients received prolonged combination antimicrobial therapy, with one patient requiring surgical debridement. Complete clinical healing was achieved in all cases.

Conclusions

*Mycobacterium fortuitum* is an important but frequently overlooked cause of chronic post-procedural wound infections. Isolation of *M. fortuitum* in seven clinically suspected NTM wound infections highlights the importance of maintaining a high index of clinical suspicion in patients with persistent non-healing post-operative wounds, particularly when routine bacterial cultures are negative or there is poor response to conventional antibacterial therapy. Early mycobacterial culture with species-level identification is essential for timely diagnosis and appropriate management.

## Introduction

Post-procedural wound infections remain a significant clinical challenge due to their often-indolent progression and resistance to empirical antibiotic regimens. These infections are commonly attributed to pyogenic bacteria; however, a subset of patients develops chronic non-healing wounds despite repeated courses of conventional antimicrobial therapy and routine wound care. Among these, nontuberculous mycobacteria (NTM), specifically *Mycobacterium fortuitum*, have emerged as formidable pathogens frequently associated with recalcitrant surgical site infections [1,2]. The organism is ubiquitous in soil, natural water sources, and biofilms and has been implicated in outbreaks linked to contaminated water systems, inadequately disinfected surgical instruments, and healthcare-associated environmental reservoirs [3,4].

These infections usually present several days to weeks after the initial procedure with persistent discharge, delayed wound healing, abscesses or sinus formation, often with minimal systemic symptoms [4]. The propensity of these organisms to form biofilms on foreign bodies, such as surgical mesh and silicone implants, complicates clinical management and often necessitates surgical debridement or device removal alongside prolonged multidrug antibiotic regimens [5].

Recent clinical observations in the region suggest a rising incidence of these infections following both laparoscopic and open surgical procedures, necessitating a shift in diagnostic protocols to include specialized acid-fast bacilli culture and molecular speciation [6,7]. Clinicians often face significant diagnostic challenges, as these infections frequently mimic common bacterial etiologies, leading to prolonged delays in identifying the causative rapidly growing mycobacteria.

Recent studies in resource-limited Indian settings have highlighted that limited diagnostic infrastructure and a general lack of clinical awareness regarding NTM contribute to underreporting and mismanagement of these surgical site infections [8]. Addressing this burden requires an integrated approach, as reliance solely on direct Ziehl-Neelsen (ZN) staining may lead to high false-negative rates and subsequent therapeutic failure [9].

Although sporadic cases and outbreaks of post-surgical infections due to rapidly growing mycobacteria have been reported from different regions of India, data from Eastern India remain limited. During a one-year period at our tertiary care centre, seven patients with post-procedural wound infections were identified with culture-confirmed *M. fortuitum*. This case series describes their clinical and microbiological characteristics and highlights the importance of maintaining clinical suspicion for rapidly growing NTM in chronic non-healing postoperative wounds.

## Materials and methods

Study design and settings

This hospital-based cross-sectional observational study was conducted in the Department of Microbiology in collaboration with the Departments of General Surgery and Obstetrics & Gynaecology at a tertiary care teaching hospital in Eastern India between September 2024 and August 2025. Patients presenting with clinically suspected chronic or persistent post-procedural wound infections were evaluated for NTM infection. Seven patients with culture-confirmed *M. fortuitum* infection were subsequently included in the present nested case series.

Ethical consideration

The study protocol was approved by the Institutional Ethics Committee. Written informed consent was obtained from all participants before enrolment. Confidentiality of patient data was strictly maintained throughout the study.

Study population

All patients fulfilling the predefined clinical criteria for suspected post-procedural NTM wound infection during the study period underwent microbiological evaluation. Patients from whom *M. fortuitum* was subsequently isolated were included in this case series.

Inclusion criteria

Patients were eligible when they developed delayed wound healing, wound breakdown, persistent discharge, inflammation, or nodules at or near a wound after a surgical or injection-related procedure and the treating team clinically suspected NTM infection.

Exclusion criteria

Patients with acute wound infections developing within seven days of surgery and responding to routine antibacterial therapy, inadequate clinical specimen volume, or incomplete clinical information were excluded.

Data collection

Demographic characteristics, comorbidities, details of the antecedent procedure, healthcare facility where the procedure was performed, anatomical site of infection, interval between the procedure and symptom onset, clinical manifestations, prior antimicrobial therapy, microbiological findings, treatment received, surgical intervention, and clinical outcomes were recorded using a predesigned case record form (Appendices).

Sample collection

Clinical specimens, including pus, aspirates, tissue biopsies, wound swabs, and sinus tract discharge, were collected aseptically from clinically involved sites. Whenever feasible, tissue or aspirated material was preferred over superficial swabs. Specimens were transported promptly to the microbiology laboratory and processed according to standard laboratory procedures [10].

Sample processing

Each specimen was initially subjected to Gram staining, ZN staining, and routine aerobic bacterial culture. Based on clinical suspicion, all samples were simultaneously processed for mycobacterial culture using the BacT/ALERT MP automated liquid culture system (bioMérieux, France) and inoculated onto Middlebrook solid medium for isolation of mycobacteria. For liquid culture, 0.5 mL of reconstituted antimicrobial supplement containing amphotericin B, nalidixic acid, polymyxin B, trimethoprim, and fosfomycin was added to each BacT/ALERT MP bottle before inoculation to minimize contamination. Processed specimens were inoculated aseptically and incubated in the BacT/ALERT system for up to six weeks. Positive liquid culture signals were subjected to Gram staining and ZN staining. Smear‑positive liquid cultures were subcultured onto Middlebrook 7H11 agar and were incubated at 25°C, 37°C, and 42°C to facilitate the detection of both rapidly and slowly growing mycobacterial species.

Growth on solid media was evaluated based on colony morphology, growth rate, pigmentation, and acid‑fast characteristics confirmed by repeat ZN staining. All isolates were tested using the MPT64 antigen immunochromatographic assay to differentiate *Mycobacterium tuberculosis* complex (MTBC) from NTM. Isolates negative for MPT64 antigen were provisionally categorized as NTM. Species‑level identification of presumptive NTM isolates was performed using matrix-assisted laser desorption/ionization time-of-flight mass spectrometry (MALDI‑TOF MS) (Bruker Daltonics GmbH, Bremen, Germany) in accordance with the manufacturer's instructions [10]. Antimicrobial susceptibility testing was not performed because the facility for standardized antimicrobial susceptibility testing of rapidly growing nontuberculous mycobacteria was not available in our laboratory during the study period.

Treatment and outcome assessment

The antimicrobial agents used in individual patients included azithromycin, doxycycline, levofloxacin, and cotrimoxazole, with injectable agents used selectively when clinically indicated. Treatment duration and combination regimens were individualized by the treating clinicians according to clinical response. Clinical outcome was assessed by resolution of wound discharge, healing of the lesion, and absence of recurrence during follow-up.

## Results

During the 12-month study period, seven patients with culture-confirmed *M. fortuitum* post-procedural wound infections were identified. Their demographic, clinical, microbiological, treatment, and outcome characteristics are summarized in Table 1. The mean age was 39 years (range, 25-58 years), and six patients (85.7%) were female. The preceding procedures included laparoscopic cholecystectomy, appendicectomy, total abdominal hysterectomy with bilateral salpingo-oophorectomy (TAH-BSO), ovarian cystectomy, and intramuscular injection. Five of the seven patients had undergone their index procedures at local nursing homes before referral to our centre. Patients presented predominantly with persistent wound discharge, delayed wound healing, sinus formation, or localized abscess formation. Direct ZN staining was positive in two cases, while all seven isolates were recovered by BacT/ALERT MP and identified as *M. fortuitum* by MALDI-TOF MS. All patients received combination antimicrobial therapy, with one requiring surgical debridement, and all achieved clinical healing during follow-up.

**Table 1:** Clinical summary of the seven cases of Mycobacterium fortuitum infection. TAH-BSO: total abdominal hysterectomy with bilateral salpingo-oophorectomy; IM: intramuscular; ZN: Ziehl-Neelsen; TTD: time to detection; AZ: azithromycin; DOXY: doxycycline; LEVO: levofloxacin; COT: cotrimoxazole; INJ MERO: meropenem; CONS: coagulase-negative staphylococci.

Variables	Case 1	Case 2	Case 3	Case 4	Case 5	Case 6	Case 7
Age (in years)/gender	38/Female	32/Female	58/Female	42/Female	38/Female	40/Female	25/Male
Comorbidities	No	No	No	Hypothyroidism	No	No	No
Prior procedure	Lap cholecystectomy	Lap cholecystectomy	Appendicectomy	Ovarian cystectomy	Ovarian cystectomy	TAH-BSO	IM injection
Index procedure done in	Local nursing home	Local nursing home	Local nursing home	Local nursing home	Private hospital	Local nursing home	Government tertiary care centre
Time to symptom onset	1 month	3 weeks	6 weeks	2 months	1 month	1 week	1 month
Symptoms	Pus discharge and pain	Multiple discharging sinus	Discharging sinus	Abscess and pus discharge	Multiple discharging sinus	Discharging sinus and pain	Discharging sinus
Duration of symptoms before diagnosis	5 months	2 months	4 months	2 years	4 months	3 months	2 months
ZN smear	Negative	Negative	Positive	Negative	Negative	Negative	Positive
BacT/ALERT TTD	7 days	11 days	7 days	4 days	5 days	5 days	6 days
Routine aerobic culture growth	No growth	*Acinetobacter* sp.	No growth	No growth	CONS	No growth	No growth
Antimicrobial therapy	AZ, DOXY, LEVO, COT	AZ, DOXY, LEVO	AZ, DOXY, LEVO	AZ, DOXY, LEVO, COT, MERO	AZ, DOXY, LEVO	AZ, DOXY, LEVO	AZ, DOXY, LEVO
Outcome	Healed	Healed	Healed	Healed	Healed	Healed	Healed

Case 1

A 38-year-old woman with no reported comorbidities presented with pain and persistent purulent discharge five months after symptoms began, which had occurred one month after laparoscopic cholecystectomy at a local nursing home. Local examination showed a non-healing discharging wound with tenderness. The ZN smear was negative, routine aerobic culture showed no growth, and the BacT/ALERT MP culture became positive after seven days. MALDI-TOF MS identified *M. fortuitum*. She received azithromycin, doxycycline, levofloxacin, and cotrimoxazole. The wound healed during follow-up.

Case 2

A 32-year-old woman with no reported comorbidities developed multiple discharging sinuses three weeks after laparoscopic cholecystectomy at a local nursing home and remained symptomatic for two months before diagnosis. Examination showed multiple draining sinus tracts at the operative site. The ZN smear was negative; routine aerobic culture grew *Acinetobacter *species. The BacT/ALERT MP culture became positive after 11 days, and MALDI-TOF MS identified *M. fortuitum*. She was treated with azithromycin, doxycycline, and levofloxacin, with complete healing during follow-up.

Case 3

A 58-year-old woman with no reported comorbidities presented with a persistent discharging sinus four months after symptoms began, following onset six weeks after appendicectomy at a local nursing home. Examination demonstrated wound dehiscence with seropurulent discharge (Figure 1A). The ZN smear was positive, routine aerobic culture showed no growth, and the BacT/ALERT MP culture became positive after seven days. MALDI-TOF MS identified *M. fortuitum*. Treatment with azithromycin, doxycycline, and levofloxacin resulted in healing (Figure 1B).

**Figure 1:**
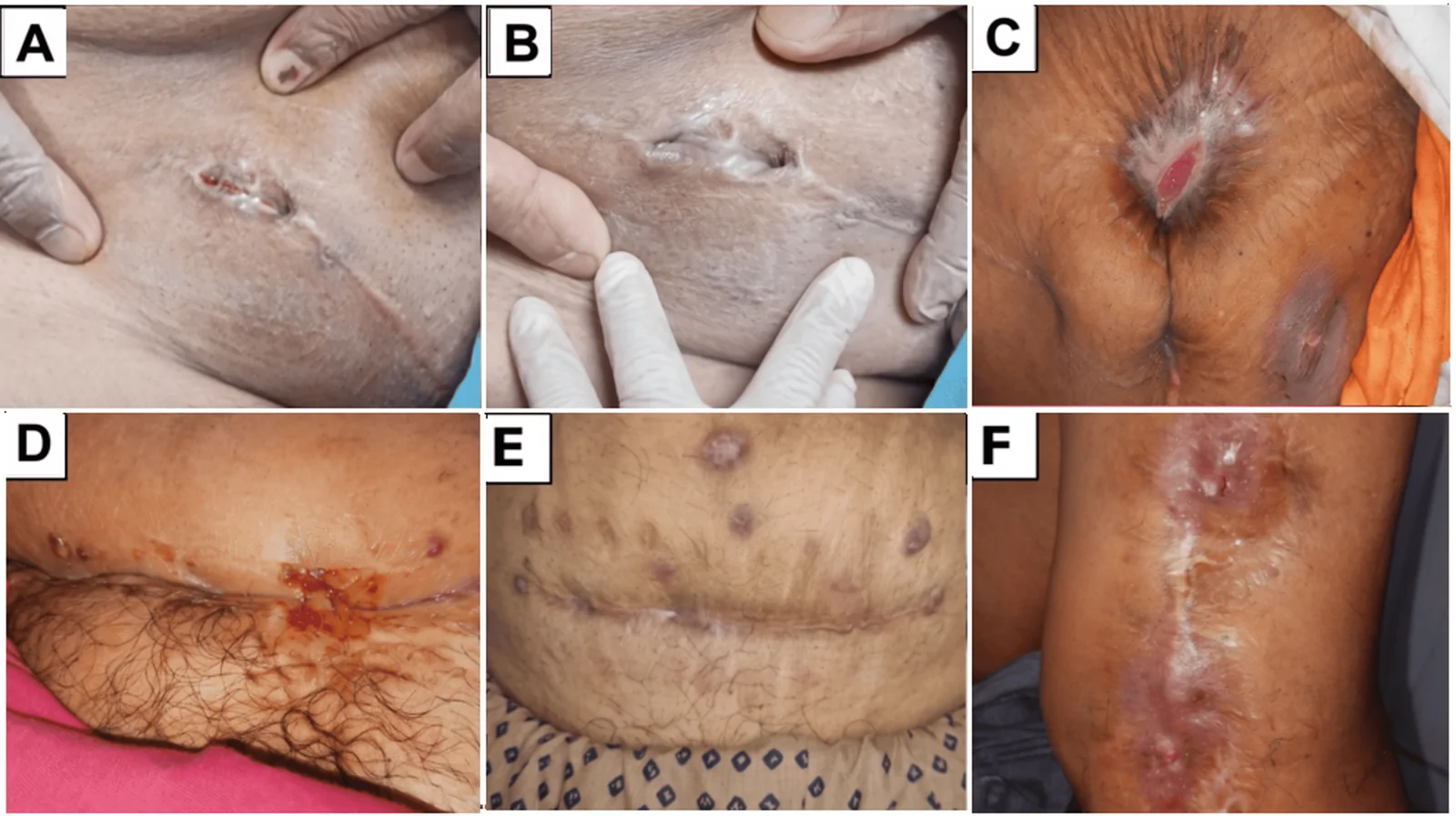
Representative clinical manifestations of post-procedural Mycobacterium fortuitum infection. (A) Persistent wound dehiscence with seropurulent discharge following appendicectomy. (B) Healed postoperative incision following combination antimicrobial therapy. (C) Chronic non-healing postoperative wound with ulceration and adjacent nodular lesions. (D) Persistent seropurulent discharge from the surgical incision following TAH-BSO. (E) Healed TAH-BSO incision following completion of prolonged combination antimicrobial therapy. (F) Multiple discharging sinuses following intramuscular injection. TAH-BSO: total abdominal hysterectomy with bilateral salpingo-oophorectomy.

Case 4

A 42-year-old woman with hypothyroidism developed an abscess with purulent discharge two months after an ovarian cystectomy at a local nursing home. Symptoms persisted for two years before diagnosis. Examination showed a chronic non-healing wound with ulceration and adjacent nodular lesions (Figure 1C). The ZN smear was negative, routine aerobic culture showed no growth, and the BacT/ALERT MP culture became positive after four days. MALDI-TOF MS identified *M. fortuitum*. She received azithromycin, doxycycline, levofloxacin, cotrimoxazole, and injectable meropenem and underwent surgical debridement. The wound healed during follow-up.

Case 5

A 38-year-old woman with no reported comorbidities developed multiple discharging sinuses one month after an ovarian cystectomy at a private hospital and had symptoms for four months before diagnosis. Examination showed multiple draining sinus tracts near the operative site. The ZN smear was negative, routine aerobic culture grew coagulase-negative staphylococci, and the BacT/ALERT MP culture became positive after five days. MALDI-TOF MS identified *M. fortuitum*. She received azithromycin, doxycycline, and levofloxacin, and the lesions healed during follow-up.

Case 6

A 40-year-old woman with no reported comorbidities presented with pain and a discharging sinus three months after symptoms began, which had started one week after TAH-BSO at a local nursing home. Examination showed persistent seropurulent discharge from the abdominal incision (Figure 1D). The ZN smear was negative, routine aerobic culture showed no growth, and the BacT/ALERT MP culture became positive after five days. MALDI-TOF MS identified *M. fortuitum*. She received azithromycin, doxycycline, and levofloxacin, with healing of the incision during follow-up (Figure 1E).

Case 7

A 25-year-old man with no reported comorbidities developed a discharging sinus one month after an intramuscular injection at a government tertiary care centre and remained symptomatic for two months before diagnosis. Examination showed multiple discharging sinuses at the injection site (Figure 1F). The ZN smear was positive, routine aerobic culture showed no growth, and the BacT/ALERT MP culture became positive after six days. MALDI-TOF MS identified *M. fortuitum*. He received azithromycin, doxycycline, and levofloxacin, and the lesion healed during follow-up.

## Discussion

Rapidly growing nontuberculous mycobacteria (RGM), particularly *M. fortuitum*, are increasingly recognized as important causes of post-procedural skin and soft tissue infections [11]. Their ubiquitous presence in environmental reservoirs and their ability to survive in healthcare-associated water systems and biofilms make them potential causes of healthcare-associated infections following surgical and other invasive procedures [3,4].

In our study, seven patients with post-procedural wound infections were diagnosed with *M. fortuitum*. Most patients presented with persistent wound discharge and delayed wound healing several weeks to months after surgery or invasive procedures. Similar presentations have been described in previous reports of rapidly growing mycobacterial infections following surgical procedures [4]. Most patients had persistent wound discharge with delayed healing several weeks to months after surgery and had received empirical antibacterial therapy before diagnosis. Similar clinical presentations have been reported in previous Indian studies, emphasizing that delayed diagnosis often results from a low index of clinical suspicion [12]. Therefore, clinicians should suspect NTM infection in any chronic postoperative wound that fails to respond to conventional antibacterial therapy, particularly when routine bacterial cultures are repeatedly negative.

One of the important microbiological observations in our study was the limited diagnostic utility of direct ZN staining. Only two of the seven culture-confirmed cases were smear-positive, demonstrating that negative microscopy cannot reliably exclude NTM infection. Similar findings have been described previously and are likely related to the low bacillary burden in localized soft tissue infections [11,13].

Consequently, mycobacterial culture remains the cornerstone of diagnosis. In our laboratory, BacT/ALERT MP liquid culture successfully recovered all isolates, while MALDI-TOF MS enabled rapid species-level identification. These findings demonstrate that timely diagnosis of post-procedural NTM infections is achievable in routine microbiology laboratories, provided clinicians communicate their suspicion, and appropriate mycobacterial investigations are requested. The combination of liquid culture with rapid identification methods can substantially reduce diagnostic delays and facilitate earlier institution of appropriate therapy [10].

The optimal management of extrapulmonary *M. fortuitum* infection remains challenging because no dedicated evidence-based guidelines specifically address postoperative skin and soft tissue infections. Current therapeutic approaches are largely derived from observational studies, expert opinion, species identification, and antimicrobial susceptibility testing [14-16].

Although antimicrobial susceptibility testing could not be performed in this study due to resource constraints, all patients showed favourable clinical outcomes following prolonged combination therapy with azithromycin, doxycycline, levofloxacin, and cotrimoxazole, with surgical debridement required in one patient. Although our observations cannot substitute for susceptibility-guided therapy, they suggest that prompt clinical recognition, species identification, and timely initiation of combination antimicrobial therapy can result in favourable outcomes even in resource-limited settings. However, antimicrobial susceptibility testing should remain the preferred approach whenever laboratory facilities are available because resistance patterns among rapidly growing mycobacteria are variable and may influence treatment success [14,16].

Notably, most patients in our series had undergone their index surgical procedure in peripheral nursing homes before presenting to our tertiary care centre with chronic non-healing wounds. Although a common source of infection could not be established in the absence of environmental sampling or molecular typing, this observation is noteworthy. Previous outbreak investigations have demonstrated that rapidly growing mycobacteria can colonize hospital water systems, inadequately disinfected surgical instruments, disinfectant solutions, and biofilm-containing moist environments [3,17]. These findings highlight the need for stringent infection prevention and control practices, particularly in smaller healthcare facilities where standardized sterilization and surveillance protocols may be less rigorously implemented. Increased awareness among surgeons and infection control teams may facilitate earlier recognition and prevention of healthcare-associated NTM infections.

Strengths

This study has several strengths. First, it represents one of the few case series from Eastern India describing microbiologically confirmed post-procedural *M. fortuitum* skin and soft tissue infections. Second, all cases were confirmed by dedicated mycobacterial culture followed by species-level identification using MALDI-TOF MS, ensuring an accurate microbiological diagnosis. Finally, the study emphasizes the pivotal role of close clinician-microbiologist collaboration and early clinical suspicion in establishing the diagnosis of chronic postoperative wound infections caused by rapidly growing mycobacteria.

Limitations

This study has several limitations. It was conducted at a single tertiary care centre and included a small number of cases, limiting the generalizability of the findings. Environmental sampling, infection-control audits, and molecular typing were not performed; therefore, a common source or epidemiological linkage between cases could not be established. Antimicrobial susceptibility testing was not performed, and treatment regimens were not standardized; consequently, clinical outcomes cannot be attributed to any specific antimicrobial regimen. Despite these limitations, the series provides clinically and microbiologically characterized examples of post-procedural *M. fortuitum* infection and emphasizes the importance of early clinical suspicion and appropriate laboratory investigation.

## Conclusions

*Mycobacterium fortuitum* should be considered in patients presenting with chronic non-healing post-procedural wounds, particularly when there is persistent discharge, delayed wound healing, or a poor response to conventional antibacterial therapy. Early mycobacterial culture and species-level identification can facilitate diagnosis and guide clinical management. Increased awareness among clinicians and effective communication with microbiology laboratories may help reduce diagnostic delays in healthcare-associated rapidly growing NTM infections.

## Appendices

**Table 2:** Case record form.

Date:	Address details:	Village & PO:
Name:		PS:
Age:		District:
Gender:		Pin:
CR No:	Mobile No:	
Occupation:	Department (OPD/Ward):	
Sample Type:	Sample site:	

Complaints	Yes/No	Duration
Discharge		
Pain		
Swelling		
Fever		
Others		

Surgery		Trauma		Injection	
Open/Laparoscopic		Type (Blunt/Sharp)		Site	
Site		Site		Place	
Place		Mode		Mode	
Post-surgical history					
Others types					

By Whom		Cleaning with proper disinfectant	
Where		Frequency	
How			

Drugs/Antibiotic	Dose	Duration

Comorbid Conditions	Yes/No	Duration
Diabetes		
Hypertension		
Chronic diseases		
Immunosuppressive drugs		
Others		
